# Tal6 From *Trichoderma atroviride* Is a LysM Effector Involved in Mycoparasitism and Plant Association

**DOI:** 10.3389/fmicb.2019.02231

**Published:** 2019-09-25

**Authors:** Yordan J. Romero-Contreras, Claudia A. Ramírez-Valdespino, Paulina Guzmán-Guzmán, Juan Ignacio Macías-Segoviano, Julio César Villagómez-Castro, Vianey Olmedo-Monfil

**Affiliations:** Departamento de Biología, División de Ciencias Naturales y Exactas, Universidad de Guanajuato, Guanajuato, Mexico

**Keywords:** endophyte, antagonism, MAMP, symbiosis, chitinases, effector

## Abstract

LysM effectors play a relevant role during the plant colonization by successful phytopathogenic fungi, since they enable them to avoid either the triggering of plant defense mechanisms or their attack effects. Tal6, a LysM protein from *Trichoderma atroviride*, is capable of binding to complex chitin. However, until now its biological function is not completely known, particularly its participation in plant–*Trichoderma* interactions. We obtained *T. atroviride Tal6* null mutant and *Tal6* overexpressing strains and determined the role played by this protein during *Trichoderma*-plant interaction and mycoparasitism. LysM effector Tal6 from *T. atroviride* protects the hyphae from chitinases by binding to chitin of the fungal cell wall, increases the fungus mycoparasitic capacity, and modulates the activation of the plant defense system. These results show that beneficial fungi also employ LysM effectors to improve their association with plants.

## Introduction

Effectors are molecules derived from microorganisms, like fungi, that participate in the establishment of its associations with other organisms, altering host structure and function (Selin et al., 2016); they were first described in pathogenic systems, playing an important role in facilitating pathogen infection or triggering host defense responses (Hogenhout et al., 2009). In beneficial fungus–plant interactions, effectors are important molecules to the establishment of symbiotic relationships, as shown by the interaction between the mycorrhiza *Laccaria bicolor* with *Populus trichocarpa*, where the effector MiSSP7 is essential to the fungus–plant root association (Plett et al., 2011), or SP7 from *Rhizophagus irregularis* (formerly, *Glomus intraradices*) that promotes the symbiosis with *Medicago truncatula* (Kloppholz et al., 2011).

Effectors are classified according to their localization in the host cell; in the case of those involved in plant-fungal associations, they could be apoplastic and cytoplasmic (Kamoun, 2006). Classification is also done according to the type of molecule or their activity, such as cerato-platanins, proteases, CFEM effectors, among others (Sharpee and Dean, 2016; Guzmán-Guzmán et al., 2017).

It has been proposed that successful phytopathogens have developed effector-based strategies to evade the plant defense responses, and to avoid being detected by the plant receptors (De Jonge and Thomma, 2009; Kombrink and Thomma, 2013). Nowadays, effectors with LysM motifs are being broadly studied due to their role in plant–pathogen interactions. The LysM motif is a carbohydrate-binding module, approximately of 50aa, that binds to *N-*acetyl-D-glucosamine (GlcNAc), the structural unit from chitin, chitosan and peptidoglycan. These effectors have a βααβ secondary structure, where both α-helices are packed on the same side of the two antiparallel β-sheet, and there can be several LysM motifs in one protein (Buist et al., 2008; De Jonge and Thomma, 2009).

In plants, the LysM motif is part of receptors involved in the recognition of GlcNAc oligosaccharides derived from different microorganisms such as phytopathogenic fungi, GlcNAc recognition triggers the activation of a signaling cascade mediated by MAP-kinases (Wan et al., 2008; Kombrink and Thomma, 2013; Meng and Zhang, 2013), involving the upregulation of several transcription factors such as the WRKY family (Pandey and Somssich, 2009), and leading to the production of defense related compounds like chitinases (Pusztahelyi, 2018).

In fungi, LysM motifs are present in two types of proteins: (i) in catalytic proteins such as subgroup C chitinases, defining enzyme architecture; and (ii) in secreted effector proteins with non-catalytic domains (De Jonge and Thomma, 2009).

According to their function, fungal LysM effectors could have three roles in plant–fungus interactions: (i) binding to fungal cell wall chitin forming a barrier, avoiding hyphal degradation by plant chitinases; (ii) binding to plant chitinases, inhibiting their activity; and (iii) hijacking GlcNAc oligomers, avoiding plant perception of fungal GlcNAc, thus preventing the triggering of plant defense responses (Sánchez-Vallet et al., 2015).

The first fungal LysM effector to be characterized was Ecp6, from *Cladosporium fulvum*. Ecp6 binds GlcNAc and contributes to *C. fulvum* virulence in tomato plants (Bolton et al., 2008). Some other LysM effectors have been identified as virulence factors like Slp1 from *Magnaporthe oryzae* (Mentlak et al., 2012); or effectors such as PeLysM1, PeLysM2, PeLysM3 and PeLysM4 from *Penicillium expansum*, which are predicted to sequester chitin oligomers or to protect hyphae (Levin et al., 2017). Other LysM effectors from organisms like *Colletotrichum higginsianum*, *Verticillium dahliae* or *Beauveria bassiana* have also been identified as virulence factors, with different number of LysM motifs, ranging from 1 motif to 5, as summarized in the Supplementary Table S1.

LysM proteins are present not just in plant pathogenic fungi or plants. Plant beneficial fungi also have LysM protein coding genes among their genomes. Such is the case of the soil-living fungal genus *Trichoderma*, which groups species that are recognized because of their ability to establish a beneficial interaction with plants, as an endophyte, and as a powerful mycoparasite of phytopathogenic fungi (Kumar et al., 2018). Several studies have shown that *Trichoderma* spp. possesses hundreds of potential effector coding genes among its genome (Schmoll et al., 2016; Guzmán-Guzmán et al., 2017; Mendoza-Mendoza et al., 2018; Nogueira-Lopez et al., 2018), which are classified according to their putative function as LysM, cerato-platanins, hydrophobins, and thioredoxins, among others.

Regarding LysM effectors, *Trichoderma atroviride* possesses eight putative LysM protein coding sequences in its genome–*Tal1*, *Tal2a*, *Tal2b*, *Tal4*, *Tal5*, *Tal6*, *Tal50*, and *Tal51*– (Gruber et al., 2011); the first six genes have effector characteristics (Guzmán-Guzmán et al., 2017). During the beneficial interaction between *T. atroviride* and the model plant *Arabidopsis thaliana*, *Tal2a* (referred to as *TaLysM1* in Guzmán-Guzmán et al., 2017) increased its expression and for Tal6 has been demonstrated the ability to bind GlcNAc compounds (Seidl-Seiboth et al., 2013); however, it is not known if Tal2a or Tal6 play a role in recognition and/or protection events during the interaction of *Trichoderma* with plants, or even with other fungi during mycoparasitism, via one or more of the functions that LysM proteins have.

In this work, because its function binding GlcNac oligomers has already been determined (Seidl-Seiboth et al., 2013), we took on the task of determining the role of Tal6 protein in the interaction of *T. atroviride* with plants and phytopathogens, in order to get a better understanding of the mechanisms controlling the *Trichoderma*’s biological associations.

## Materials and Methods

### Biological Material

*Trichoderma atroviride* IMI206040 was used in this study and from that strain we generated the *Tal6* mutant strains using the Double Joint PCR protocol (Yu et al., 2004), which consists in performing three rounds of PCR to generate an interruption cassette containing the gene *hph* (hygromycin phosphotransferase, that confers resistance to the antibiotic hygromycin) flanked by the upstream and downstream regions of the *Tal6* gene. After generating the corresponding interrupting cassette, we followed the protocol reported by Castellanos et al. (2010) to obtain and transform protoplasts from *T. atroviride*, using 20 μg of the cassette. Transformants were verified using primers flanking the interruption cassette (Supplementary Figure S1), to make sure the deletion was successful, and were subjected to single spore selection. After four single spore rounds, no wild type gene amplification was detected by real-time reverse transcription (qRT-PCR) (Supplementary Figure S2), indicating that the deletion of the gene was successful. Null mutant strains were maintained all the time on PDA selective medium.

To generate the *Tal6* overexpressing (OE) strain, we used the expression vector pUE08 (Esquivel-Naranjo and Herrera-Estrella, 2007) which contains the selection marker *hph* under the constitutive promoter from the *pki* gene (pyruvate kinase gen) from *Trichoderma reesei*. The ORF from the gene *Tal6* was amplified and cloned into the *Not*I and *Hin*dIII sites of the pUE08 plasmid, using the primers *Tal6*OE-F (5′-GCGGCCGCGAAAATTCCTGATCACGAATGTG-3′) and *Tal6*OE-R (AAGCTTAATTTGTATGTGGTTATTCATTTTGC), obtaining a fragment of 2721 bp. We verified the overexpression after four single spore rounds by qRT-PCR using 200 ng of cDNA and 40 amplification cycles (Supplementary Figure S2), using the glyceraldehyde-3-phosphatedehydrogenase gene (*gpd*) as control of expression (Carreras-Villaseñor et al., 2013). All fungal and plant material used in this work are described in the Supplementary Table S2.

### *Trichoderma–A. thaliana* Interactions

*Arabidopsis thaliana* Col-0 seeds were surface sterilized with absolute ethanol five times, the ethanol was decanted and the seeds were placed in 0.2X Murashige-Skoog (MS) agar plates supplemented with 100 mM MES buffer to maintain pH at 5.5 and covered with a sheet of cellophane. Plates were placed at 4°C for 48 h for vernalization and then were incubated at 24°C in a plant growth chamber (MRClab, model PGI-500 H) with 16 h/8 h light/dark cycles. After 4 days of germination, 1 × 10^6^ conidia/mL from the wild type, null mutant and OE strains were inoculated opposite to the plants. Plants and/or mycelia were collected at 2, 3, and 5 days of interaction. Plants of *Arabidopsis* were grown alone as control. Samples were frozen and kept under −70°C until further analysis.

### *Trichoderma–Solanum lycopersicum* Interactions

*Solanum lycopersicum* seeds were placed in 50 mL Falcon tubes and washed three times with a 3% sodium hypochlorite, and with a final wash of ethanol. Seeds were germinated in hydrated vermiculite and roots of tomato plants of 3 days old were incubated in 1 × 10^6^ conidia/mL from each of the *Trichoderma* strains, and in water as a control, for 90 min at room temperature. Plants were then put in plastic recipients containing 100 g of a mixture of soil, vermiculite, and perlite (3:1:1) and kept in environmental conditions with 16 h/8 h light/dark cycles and with 2 weekly irrigations of 200 mL of non-sterile water. After 2 weeks, 1 × 10^6^ conidia/g of soil from each *Trichoderma* strain were added to the soil. For the tripartite interactions, after 2 weeks of inoculation with each *Trichoderma* strain, 1 g of dry sclerotia, containing approximately 200 sclerotia from *Rhizoctonia solani* AG2, per 100 g of soil was inoculated, watering every third day. The interactions were carried out until the plants were 9 weeks old. After that, we measured root and stem length, fresh weight and plant survival. We performed two biological replicas, each one consisted of 8 plants for the control treatments (fungi-free plants or plants grown only with one of the fungi) and 12 plants for the tripartite interactions. To determine the percentage of plant survival, we considered the plants from both biological replicas as 100% for each treatment.

### Mycoparasitism Assays

Direct confrontation assays were carried out with the wild type, OE and null mutant strains from *T. atroviride*, with the phytopathogens: *Botrytis cinerea*, *Sclerotium cepivorum*, *R. solani* AG2 and *Colletotrichum lindemuthianum*. In plates containing PDA medium, a plug of mycelium was placed of each of the *T. atroviride* strains at one end of the plate, and at the other end, at approximately 5 cm from *Trichoderma*, a plug of mycelia from the phytopathogen was placed. The plates were kept in an incubator (INO 650M) at 28°C, for a period of 10 days.

### Protoplasts Formation and Cell Wall Integrity Assay

To obtain the protoplasts from *T. atroviride*, each strain was inoculated in potato dextrose broth (PDB), using 1 × 10^6^ conidia/mL and incubated at 28°C for 12–15 h, obtaining germinules. We used 15 mg/mL of *T. harzianum* enzymatic extract (Glucanex Sigma L141) 1 mL of osmotic solution (CaCl 20 Mm, Manitol 0.5 M, MES 50 Mm, pH 5.5) (Castellanos et al., 2010) and we modified the original protocol by adding 0.1 μg/mL of *Streptomyces griseus* chitinase (Sigma C-1525). The formation of protoplasts was evaluated at 15, 25, and 45 min. To recover the protoplasts, the suspension was filtered using miracloth filters and washed with 2 mL of osmotic solution. Subsequently, protoplasts were quantified in a Neubauber chamber. To observe the integrity of the cell wall in each *Trichoderma* strain, we added calcofluor white dye to a concentration of 0.1% (p/v) (Sigma 18909) (Arango and Castaneda, 1995), previous to protoplasts recovering and samples of them were observed in a fluorescence microscope (Leica DM100).

### Interference With Chitinase Activity Assay

We evaluated the activity of the chitinase from *S. griseus* (Sigma C-1525) and its inhibition by the supernatants of wild type, *Tal6-*OE1.1 and *Tal6-*Δ4.2 null mutant strains from *T. atroviride* cultures, under different conditions (Supplementary Table S3), monitoring the hydrolysis of the substrate 4-methylumberiferone chitotriose [4MU(Ch)_3_] (Kuranda and Robbins, 1987). To obtain the supernatants, we inoculated 10 mL of PDB medium with 1 × 10^6^ conidia/mL and then incubated at 28°C for a period of 12–15 min. A reaction mixture containing 20 μL of supernatant of the *T. atroviride* strains, 10 μM of 4-MU(Ch)_3_, 0.1 U/mL of chitinase from *S. griseus* and 100 mM citrate buffer (Karal, 1002) in a total volume of 100 μL. The mix was incubated at 28°C for 15 and 30 min. Subsequently, the reaction was diluted with 2.9 mL of stop solution (0.1 M NaHCO_3_ pH 10.4). For the competition conditions with GlcNAc, a preincubation of the supernatants of *Trichoderma* strains with GlcNac was carried out for 15 min at 28°C, then the chitinase enzyme and the substrate 4-MU(Ch)_3_ were added. The 4-MU released was evaluated at 15 and 30 min in luminescence spectrophotometer (Perkin-Elmer LS55) (modified from Villagómez-Castro et al., 1992).

### Bioinformatic Analysis

To carry out the comparison of the LysM motifs of the Tal6 protein with those present in effectors from fungal pathogens, we used the amino acid sequence of each motif, as reported for each pathogen listed in Supplementary Table S1. The sequences of the LysM motifs from *T. atroviride* were taken from the https://genome.jgi.doe.gov portal database. The edition of the motifs to match the number of amino acids for each of the motifs was made using the software BioEdit Sequence Alignment Editor (Hall, 1999), identifying the amino acid Cys as reference, because it is the conserved amino acid among the motifs. The alignment of the LysM motifs was done through the platform MAFFT alignment https://mafft.cbrc.jp/alignment/server/, which performs the alignment in a concise and quick way. Subsequently, the phylogenetic tree was constructed using the free software MEGA7 (Molecular Evolutionary Genetics Analysis), using the ClustalW algorithm with 1500 bootstrap, and the neighbor joining method. To analyze the conserved amino acids between each one of the LysM motifs from the effectors, the WebLOGO platform^1^ was used.

### DNA Extraction

Fungal DNA was extracted following the protocol reported by Raeder and Broda (1985).

### RNA Extraction and cDNA Synthesis

The collected mycelia from the mycoparasitism (before - T1-, during -T2- and after -T3- the contact with the phytopathogenic fungi) and plant interactions and the plant tissue collected from the *Trichoderma–*plant interactions (at 2, 3, and 5 days of interaction with each *Trichoderma* strain; T1, T2, T3, respectively) were frozen immediately in liquid nitrogen. Mycelia and plant tissue were ground to a fine powder under liquid nitrogen and total RNA was isolated using the TRizol method. cDNA was synthesized with RevertAid H Minus First Strand cDNA Synthesis Kit^®^ (Thermo Fisher Scientific), following the manufacturer’s recommendations.

### Gene Expression Analysis

Primers were designed using the IDT platform^2^, considering primer size preferably 20–24 nt in length, similar melting temperatures for each primer pair, and above 60% of GC. Primers to be used in qRT-PCR reactions were designed to produce amplicons of a maximum of 200 bp length. See Supplementary Table S4 for the complete list of primers used in his work and the size for each amplification fragment. qRT-PCR reactions were performed using Fast SYBR Green Master Mix^®^ (Applied Biosystems) with 200 ng of cDNA as template. The *gpd* or the actin (*act11*) genes were used as housekeeping genes. Three technical replicates were analyzed for each type of interaction. The data were analyzed with the 2^–ΔΔCt^ method using the StepOne software (Applied Biosystems) to determine the expression of the selected genes.

### Statistical Analysis

To analyze the effect of the wild type, mutant and OE strains over the tomato plants, a one-way ANOVA for non-parametric data and Bonferroni *post hoc* test was performed, using a significance value of *p* ≤ 0.05. To qRT-PCR assays a Kruskal–Wallis and a Dunn’s *post hoc* test was carried out to compare the data obtained from the assays. Experiments were done by duplicate with three technical replicates.

## Results

### *T. atroviride* Detects Distantly the Presence of *A. thaliana* and *Tal6* Expression Is Up-Regulated in the Fungus

**FIGURE 1:**
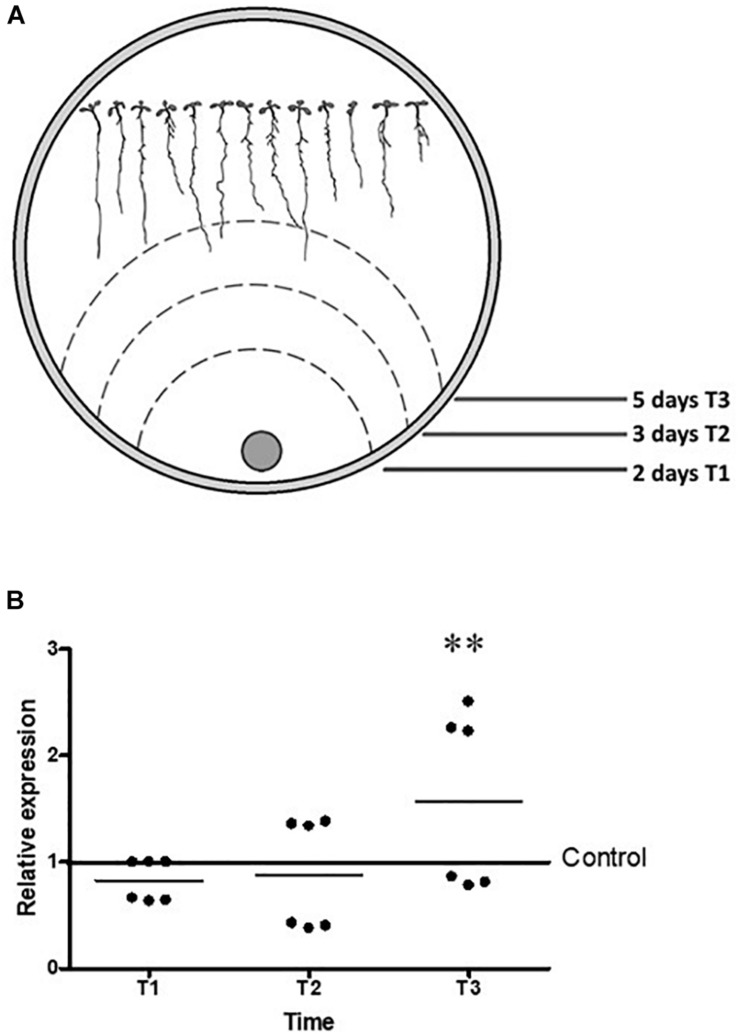
The expression of *Tal6* in *T. atroviride* is up regulated in the presence of *A. thaliana*. **(A)** Representative cartoon of *Arabidopsis* seedlings that were inoculated with *T. atroviride*, T1, T2, and T3 represents mycelia growth at three times. **(B)** qRT-PCR expression assay of *Tal6* in interaction with *Arabidopsis*. Mycelia was collected at T1, T2, and T3. Glyceraldehyde phosphate dehydrogenase gene (*gpd*) was used as endogenous expression control. Beeswarm scatter plot shows the results obtained of two biological replicates with three technical replicates. Control line represents the normalized expression of *Tal6* without the presence of *Arabidopsis.* Data were analyzed using an ANOVA non-parametric test. Asterisks represent statistical differences between the control condition and the treatments. ^∗^*p* < 0.05.

We performed a bioinformatic analysis using the JGI database^3^ to determine the number of LysM motifs contained in each of the six genes reported as putative effectors for *T. atroviride*. The six genes sequences have a signal peptide, an RGYR translocation motif, and different number of LysM motifs (Supplementary Figure S3). The Tal6 protein has 7 LysM motifs, corroborating previous analysis (Seidl-Seiboth et al., 2013), being the gene with the most motifs among the six analyzed. Preliminary, we determined the differential expression of the six genes during the interaction with *A. thaliana* by RT-PCR, observing expression for genes *Tal2a*, *Tal2b*, *Tal4*, and *Tal6* while for the genes *Tal1* and *Tal5* we did not find any expression, so we consider them not be active during the conditions we tested (data not shown). We corroborated the expression of *Tal6* during the interaction with *A. thaliana* at 2, 3, and 5 days of interaction, corresponding to T1, T2, and T3 of collect time (Figure 1A), by qRT-PCR and we observed that *Tal6* was differentially up-regulated in the presence of *A. thaliana* at the three times collected, even before of a physical contact between roots and mycelia, with a relative expression of two times fold compared to the control (Figure 1B), suggesting that *Tal6* could have a role in the establishment of the *Trichoderma–*plant interaction.

### Tal6 Plays a Role in Protecting the *T. atroviride* Hyphae Against Chitinases and Enhances Its Mycoparasitic Activity

To elucidate the biological function of the product coded by the gene *Tal6*, we carried out several experiments in order to determine if one of the functions proposed for LysM effectors were true for Tal6. We generated a *Tal6* null mutant strain, *Tal6-*Δ4.2, and an overexpressing strain, *Tal6-*OE1.1. After confirming the absence of *Tal6* mRNA in the *Tal6-*Δ4.2 null mutant and its overproduction in the *Tal6-*OE1.1 strain by qRT-PCR (Supplementary Figure S2), we proceeded to design the experiments to characterize these strains.

**FIGURE 2:**
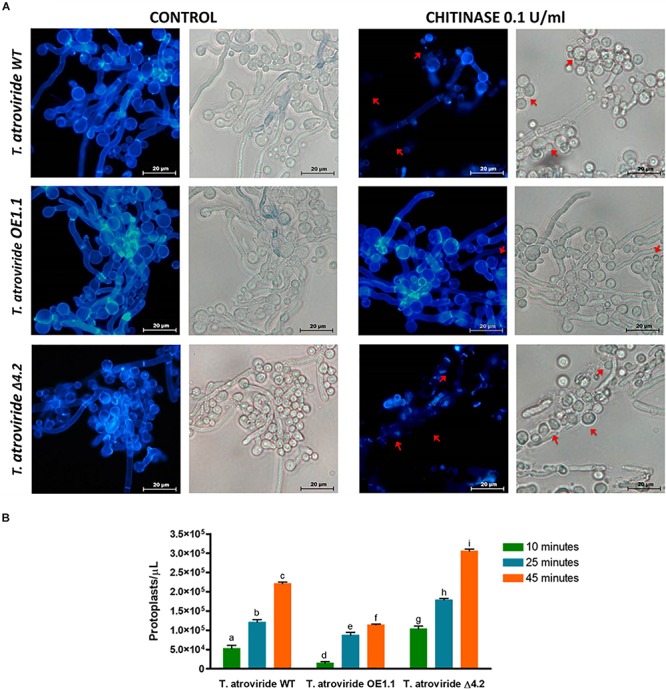
Chitinases susceptibility tests and protoplasts formation of the *T. atroviride* wild type, *Tal6-*Δ4.2 and *Tal6-*OE1.1 strains. **(A)** Control panel shows the hyphae integrity, marked by the white calcofluor dye in the cell wall chitin. Chitinase panel shows the damage of the cell wall chitin of each strain after treatment with 15 mg/mL of *T. harzianum* enzymatic cocktail and 0.1 U/mL of *S. griseus* chitinase, red arrows indicate the presence of protoplasts, structures that lack cell wall. **(B)** Protoplast formation from *T. atroviride* wild type, *Tal6-*Δ4.2 and *Tal6-*OE1.1 strains after 15, 25, and 45 min of incubation with 15 mg/mL of *T. harzianum* enzymatic cocktail and 0.1 U/mL of *S. griseus* chitinase. Data from three independent experiments were analyzed with a two-way ANOVA and a Bonferroni *post hoc* test to compare all treatments; *p* < 0.001. Letters indicate statistically differences between treatments and strains.

To determine the resistance/susceptibility of the wild type, *Tal6-*Δ4.2 and *Tal6-*OE1.1 strains to chitinases, we used calcofluor white dye, which binds to the chitin, evidencing the presence of chitin in the fungal cell wall. We observed complete integrity of the hyphae from each *Trichoderma* strain under control condition, without enzyme (Figure 2A). When the enzymatic cocktail, enriched with chitinase, was added to the hyphae both wild type and *Tal6-*Δ4.2 strains show protoplast formation (Figure 2A, upper and lower rows), characterized by the partial or total absence of cell wall, where the calcofluor white fluorescent signal is less visible or absent (red arrows), showing a slightly higher stain in the wild type strain than in the null mutant. Whereas, the *Tal6-*OE1.1 strain still shows a strong fluorescent signal, compared to the other strains (middle row, Figure 2A), although we can see some protoplasts too (red arrows). We quantified the protoplasts formed in each strain after treatment with lytic enzymes, observing that each strain release protoplasts at the times tested, and the *Tal6-*Δ4.2 strain formed more protoplasts at the three times measured compared to the other strains, and the *Tal6-*OE1.1 strain showed the least quantity of protoplasts formed (Figure 2B). These results suggest that Tal6 is involved in the resistance against chitinases activity, protecting *T. atroviride* from chitinases of its hosts.

### Tal6 Increases the Antagonistic Capacity of *T. atrovirid*e

During a mycoparasitic interaction, participants produce several extracellular enzymes (chitinases, glucanases, proteases) in order to degrade the host cell wall favoring the hyphal penetration by the mycoparasite, and to defend the host against the attacker. *Trichoderma* species are exceptional and effective mycoparasites, due to their capacity to secrete a plethora of cell wall degrading enzymes (Kubicek et al., 2011; Gruber and Seidl-Seiboth, 2012; Mendoza-Mendoza et al., 2018). Being such a mycoparasite, in turn *Trichoderma* must need to defend itself from the host’s hydrolytic enzymes. To determine if the product coded by the gene *Tal6*, could be protecting the hyphae during a mycoparasitic interaction, we first determined if this gene is expressed during such conditions.

**FIGURE 3:**
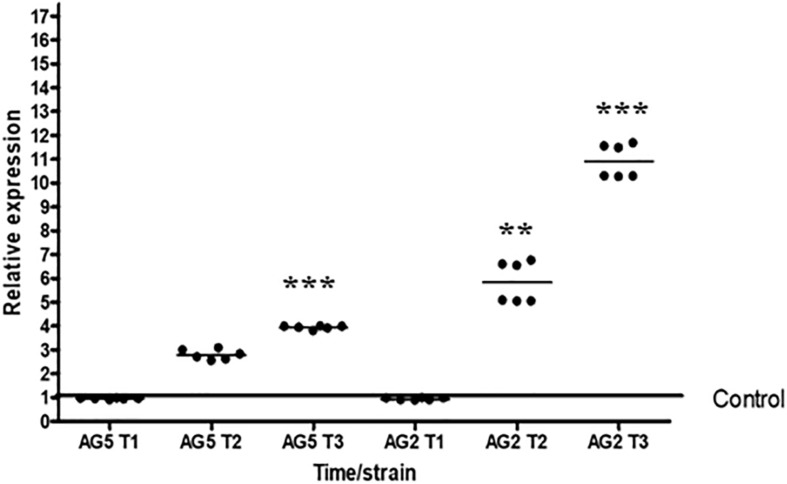
Expression of *Tal6* is affected in the presence of phytopathogens. qRT-PCR expression assay of *Tal6* in *T. atroviride* wild type in confrontation with *R. solani* AG2 and AG5. Mycelia was collected before the contact (T1), during the contact (T2) and after the contact (T3). Mycelia of *Trichoderma* cultured alone was used as control. Glyceraldehyde phosphate dehydrogenase gene (*gpd*) was used as endogenous expression control. Beeswarm scatter plot shows the results obtained of two biological replicates with three technical replicates. Control line represents the normalized expression of *Tal6* without the presence of phytopathogens. Data were analyzed using an ANOVA non-parametric test. Asterisks represent statistical differences between the control condition and the treatments. ^∗∗^*p* < 0.01 and ^∗∗∗^ *p* < 0.001.

We determined the expression level of the gene *Tal6* by qRT-PCR during confrontation with the phytopathogen *R. solani* anastomosis groups AG2 and AG5. We observed that *Tal6* was expressed at all times tested, but its expression increases differentially during and after contact with both *R. solani* anastomosis groups. Although its expression level is higher in confrontation with *R. solani* AG2, showing approximately 10 times more of relative expression after contact with the pathogen (*p* < 0.001, Figure 3), and 4.5 times during contact (*p* < 0.001, Figure 3). This result indicates that *Tal6* could be also involved in mycoparasitic interactions.

**FIGURE 4:**
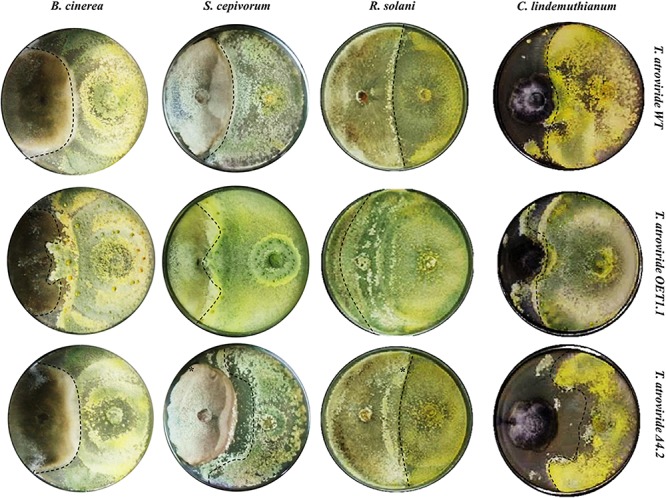
Mycoparasitic capacity of *T. atroviride* wild type, *Tal6-*Δ4.2 and *Tal6-*OE1.1 strains against different phytopathogens. PDA plates at 10 days of confrontation between each *Trichoderma* strain with *B. cinerea*, *S. cepivorum*, *R. solani* AG2 and *C. lindemuthianum.* Dotted lines show the growth of each *Trichoderma* strain.

**FIGURE 5:**
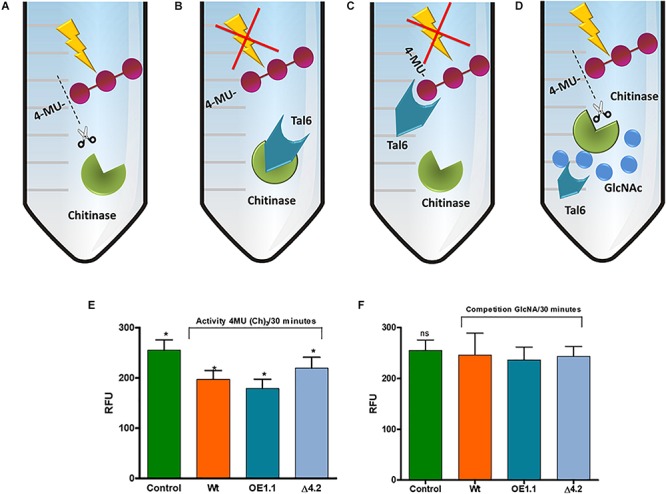
Tal6 binds GlcNAc oligomers. **(A–D)** Represents possible *in vitro* reactions. **(A)** Chitinase from *S. griseus* uses 4-MU(Ch)_3_ as substrate, releasing the three GlcNAc units. **(B)** Tal6 inhibits the chitinase activity, preventing 4-MU(Ch)_3_ hydrolysis. **(C)** Tal6 binds to the three GlcNAc units, preventing the chitinase from acting on its substrate. **(D)** In a substrate competition, Tal6 binds to GlcNAc monomers, allowing the chitinase to act on its substrate 4-MU(Ch)_3_. **(E)** *S. griseus* chitinase activity over 4-MU(Ch)_3_ after 30 min of incubation with each of the supernatants from *T. atroviride* wild type, *Tal6-*Δ4.2 and *Tal6-*OE1.1 strains, and without supernatant as a control condition. **(F)** *S. griseus* chitinase activity over 4-MU(Ch)_3_ after 30 min of incubation with each of the supernatants from *T. atroviride* wild type, *Tal6-*Δ4.2 and *Tal6-*OE1.1 strains and GlcNAc monomers as competitive substrate. Data were analyzed with a one-way ANOVA; asterisks represent statically differences between treatment and control conditions; ^∗^*p* < 0.05.

Along with these results, we decided to test the mycoparasitic capacity of the *Tal6-*Δ4.2 and *Tal6-*OE1.1 strains in dual cultures against phytopathogens: *B. cinerea*, *S. cepivorum*, *C. lindemutianum*, and *R. solani* AG2. Figure 4 shows that *Tal6-*OE1.1 strain overgrows each of its hosts, far better than the wild type and the null mutant strains. Meanwhile, the *Tal6-*Δ4.2 strain shows less colony growth over its hosts compared to the wild type strain, except in confrontation with *B. cinerea*, where there is no apparent difference, as shown in Figure 5 by the dotted lines. These results, altogether, show that Tal6 from *T. atroviride* could play a role in increasing the fungus mycoparasitic capacity when encountered with several phytopathogens.

### Tal6 Protects Hyphae From Chitinase Activity by Binding to Chitin in Cell Walls and Not by Inactivation of Chitinases

So far, our results indicate that Tal6 protein protects hyphae, but it remains to be determined whether it does so by binding to chitin or by inactivating chitinases. We carried out the *in vitro* quantification of chitinase activity, where the supernatants of each *Trichoderma* strain were incubated with a chitinase from *S. griseus* and the substrate 4-MU(Ch)_3_, which is a compound that has fluorescent potential, limited by the union of three units of GlcNAc in the β-(1-4) links; after enzymatic hydrolysis by chitinases, it is possible to quantify by fluorescence the amount of hydrolyzed substrate and relate it to the chitinase activity (Villagómez-Castro et al., 1992).

The *Trichoderma* supernatants, the chitinase and the substrate were incubated for 30 min and a control condition was included, corresponding to the 100% of the hydrolytic activity, in which the chitinase was incubated with the substrate 4-MU(Ch)_3_ without *Trichoderma* supernatants (Figure 5A).

We first considered the possibility that the Tal6 protein present in the supernatant inhibits the chitinase activity by direct binding to it (Figure 5B). We observed a hydrolytic activity of 77% for the wild type strain, meanwhile, for the *Tal6-*Δ4.2 strain we detected an activity of 90%, very similar to the control condition; this effect was opposite when we used the supernatants of *Tal6-*OE1.1 strain, which showed a hydrolytic activity of 54% (Figure 5E).

Another explanation for the diminishing in the hydrolytic activity that we observed in the OE supernatant is that the Tal6 protein binds to the three GlcNac units of the substrate 4-MU(Ch)_3_, preventing the chitinase from hydrolyzing it (Figure 5C). To prove this, we decided to pre-incubate the supernatants of the strains with GlcNac to recreate a substrate competition event (Figure 5D); after that, the chitinase and the substrate were added and incubated for 30 min. The results showed 90% of chitinase activity for each of the treatments (Figure 5F), meaning that Tal6 binds to *N-*acetylglucosamine, and not to the three units of GlcNAc of the substrate. So, the diminishing in enzymatic activity that we first observed was due to the Tal6 protein binding to the GlcNAc units of 4-MU(Ch)_3_, and not to the chitinase. This suggests that Tal6 protects hyphae by binding to chitin oligomers rather than directly inhibiting the chitinase function.

### Tal6 Prevents Plant Defense Responses by Hijacking GlcNAc Oligomers

In a plant–microbe interaction, plants recognize molecules derived from the microbes, known as Microorganism-Associated Molecular Patterns (MAMPs), by binding of compounds like GlcNac to specific receptors (Wan et al., 2008). A plant disease resistance signaling cascade is activated upon recognition of chitin-derived MAMPs leading to the expression of pathogenesis related genes, as it is the case of chitinases that play a role in defense and metabolite synthesis (Meng and Zhang, 2013).

To test if Tal6 could be sequestering GlcNAc oligomers and so avoiding plant recognition of chitin derived MAMPs, we determined by semiquantitative RT-PCR the expression level of several genes related to the production of plant chitinases: two MAP-kinases involved in defense gene activation: *mpk3* and *mpk6* (Zhang and Klessig, 2001; Meng and Zhang, 2013); five WRKY transcription factors that regulate the expression of defense-related genes: *wrky22*, *wrky25*, *wrky33*, *wrky53*, and *wrky70* (Zheng et al., 2006; Pandey and Somssich, 2009); and five class IV chitinases, which are involved in defense upon fungal infection: *chitIV-1*, *chitIV-2*, *chitIV-3*, *chitIV-4*, and *chitIV-5* (Naumann and Price, 2012), using *actin2* as endogenous control.

To analyze the expression of the genes mentioned above, interaction assays were carried on between *A. thaliana* plants and the *T. atroviride* wild type, *Tal6-*Δ4.2 and *Tal6-*OE1.1 strains during 2, 3, and 5 days (Figure 1A), including *Arabidopsis* plants growing alone as a control. We observed no expression whatsoever for the genes *wrky25*, *wrky53*, and *chitIV-2* in any condition tested. Also, we did not observe significant differences in the expression level of the genes *mpk3*, *mpk6*, *wrky22*, *wrky70*, *chitIV-3*, *chitIV-4*, and *chitIV-5*, when compared to the control. But we detected a significant difference for the genes *wrky33* and *chitIV-1* (data not shown).

**FIGURE 6:**
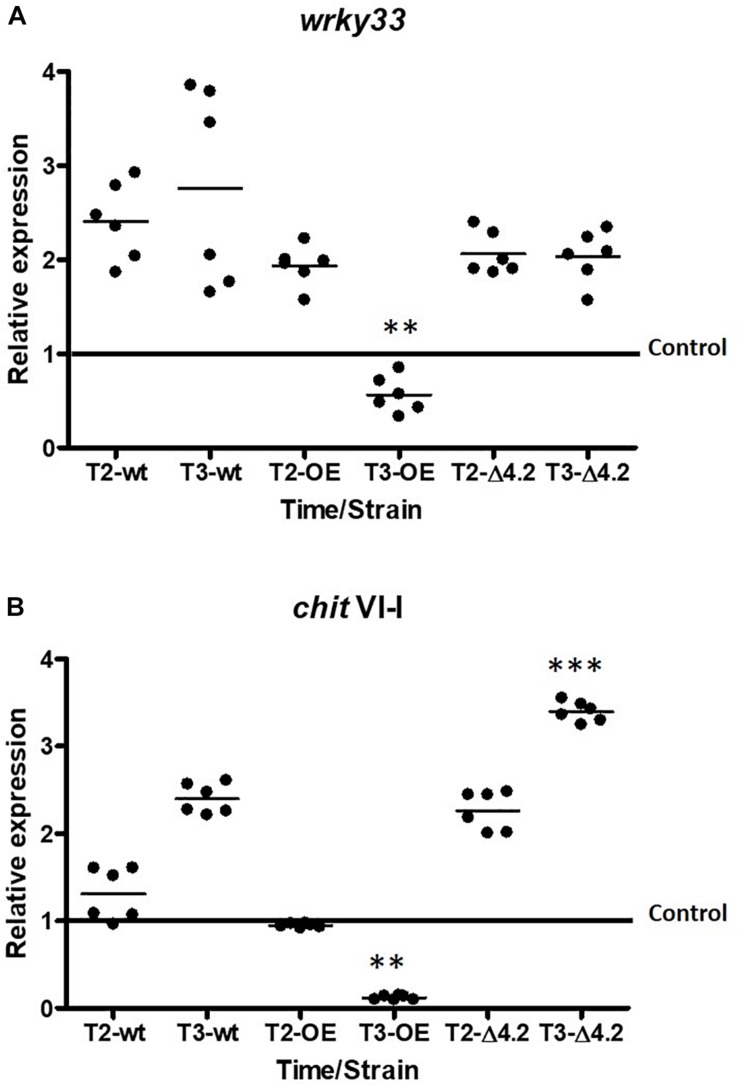
*Ta*l6 has an effect hijacking GlcNAc, avoiding the recognition mediated by plant receptors in *A. thaliana.* qRT-PCR expression assay of *wrky33* **(A)** and *chitinaseV-1* **(B)** from *A. thaliana* at 3 and 5 days post inoculation (T2 and T3, respectively). Plants cultured alone were used as control. Actin gene (*act11*) was used as endogenous expression control. Beeswarm scatter plot shows the results obtained of two biological replicates with three technical replicates. Control line represents the normalized expression of *wrky33* or *chit* VI-I without the presence of *Trichoderma* strains. Data were analyzed using an ANOVA non-parametric test. Asterisks represent statistical differences between the control condition and the treatments. ^∗∗^*p* < 0.01 and ^∗∗∗^*p* < 0.001.

To corroborate the previous observations, qRT-PCR analysis was carried out for both genes differentially expressed (Figure 6). The transcription factor *wrky33* showed a significantly decrease in its expression level at the fifth day of interaction (T3) in the presence of the *Tal6-*OE1.1 strain of 0.5 times fold, compared to the control (*p* < 0.01; Figure 6A), while its expression level was up-regulated of 2–2.5 times fold compared to the control at the T2 and T3 in the presence of the wild type and *Tal6-*Δ4.2 mutant strains (Figure 6A). A similar pattern of expression was observed for the gene *chitIV-1*, its expression diminished considerably at the T3 of interaction with the *Tal6-*OE1.1 strain, compared to the control (*p* < 0.001, Figure 6B), while it was increased in interaction with the *Tal6-*Δ4.2 mutant and wild type strains. Results regarding chitIV-1 expression in *Tal6*-Δ4.2 support the notion that the effector Tal6 interferes with the plant perception of *N-*acetylglucosamine, avoiding the activation of the signaling cascade that leads to the synthesis of defense related compounds such as chitinases.

### Comparative Analysis of LysM Domains From the Effector Tal6: LysM Domains Separate Into Two Functional Groups

**FIGURE 7:**
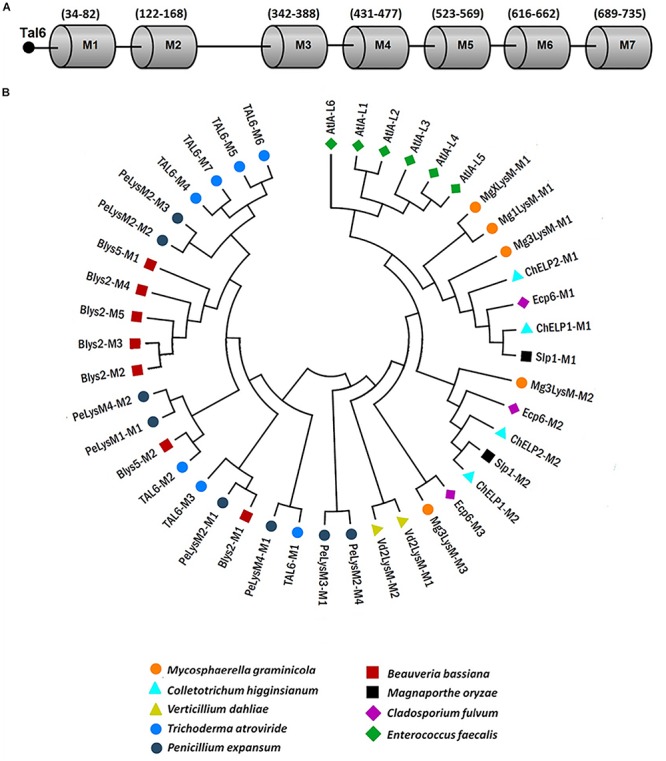
Phylogenetic relationships between Tal6 LysM motifs and LysM motifs from other fungal effectors. **(A)** Distribution of the seven LysM motifs from Tal6. **(B)** Phylogenetic tree showing the relationship of Tal6 LysM motifs and other LysM motifs from reported LysM effectors.

Tal6 effector possesses 7 LysM domains of 21 amino acid in length, approximately (Figure 7A). So far, our results suggest that, during the interaction with the plant *A. thaliana*, Tal6 from *T. atroviride* protects their hyphae from the plant hydrolytic activity, by binding to complex chitin in the cell wall, and also sequesters *N-*acetylglucosamine, thus preventing the fungus from being recognized by the plant. To determine if the functions we proposed Tal6 has are related to its LysM domain structure, we carried out a comparative analysis of the amino acid sequences of each of the seven LysM domains against the LysM effectors from other fungi that had already been characterized (Supplementary Table S1), using the program MAFFT (Multiple Alignment using Fast Fourier Transform). Besides, a phylogenetic tree was created with MEGA 7 using 1500 bootstraps in order to determine any relationship between the domains and predict the function for every effector considered. Finally, we made an analysis using the program WebLOGO to determine the similarity between the amino acid sequences from the LysM motifs, for each of the effectors. Figure 7 shows the distribution of the seven LysM domains from Tal6 (Figure 7A) and the phylogenetic relationships among the LysM domains from Tal6 and the other effectors that were compared in the analysis, along with their predicted function (Figure 7B). To our analysis, we used as an external group the effectors from the bacterium *Enterococcus faecalis*, that bind peptidoglycan and maintain a relationship with GlcNAc binding effectors, and represents the beginning of the phylogenetic tree.

Our results show that the LysM domains from *Mycosphaerella graminicola*, *C. higginsianum*, *C. fulvum* and *M. oryzae* share the most similarity between them (Figure 7B). These effectors have been shown to sequester GlcNac oligomers. The third clade, groups effectors of *V*. *dhaliae*, *M. graminicola*, and *C. fulvum*. And the last clade, groups the Tal6 M4-M7 domains, which were reported (Seidl-Seiboth et al., 2013) that can bind only complex forms of chitin, using heterologous purified proteins.

However, motifs M4-M7 of Tal6 are closely related to motifs M2 and M3 of PeLysM2 from *P. expansum.* Regarding motifs M1-M3 of Tal6, motif M1 is closely related to motif M1 of PeLysM4 from *P. expansum*; motif M2 is related to motif M2 of Blys5 from *B. bassiana*; and motif M3 is related to motifs M1 of PeLysM2 and Blys2. The topology provided by this tree highlights that the domains present in Tal6 remain in separate groups, which could be linked with two distinctive functions, although, both related to carbohydrate binding (Figure 7B). On the other hand, LogoSequence analyzes revealed that cysteine (C) is a highly conserved amino acid among the sequences of LysM motifs in fungi (Supplementary Figure S4). We also observed that other amino acids were highly conserved between the effectors, such as aspartate (Asp or D) and asparagine (Asn or N) (Supplementary Figure S4), the presence of these amino acids have been reported in the LysM domains from plant receptors, which could indicate a supposed evolutionary relationship (Buist et al., 2008; Gruber et al., 2011).

### *Tal*6 Improves Plant Fitness Against *R. solani* in Tomato Plants

Since, we found that Tal6 is involved in the *A. thaliana*–*T. atroviride* interaction, protecting the fungus against the plant hydrolytic activity and preventing the plant from detecting *Trichoderma* derived chitin fragments; and also, Tal6 plays a role in mycoparasitism increasing the fungus antagonistic capacity, we decided to determine the role of this protein in plants, when encountered with a phytopathogen like *R. solani*, using *T. atroviride Tal6-*OE1.1, *Tal6-*Δ4.2 mutant and wild type strains, and an economically important plant, like tomato.

**FIGURE 8:**
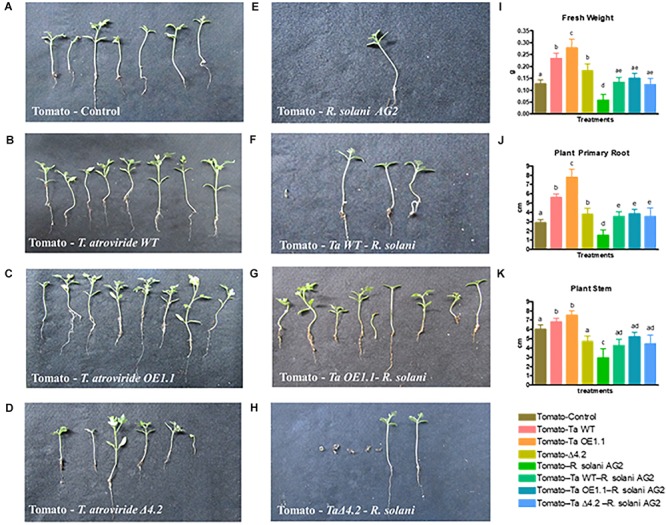
Effect of *T. atroviride* wild type, *Tal6-*Δ4.2 and *Tal6-*OE1.1 strains on tomato plants infected with *R. solani* AG2. **(A)** Tomato plants growing alone. **(B–D)** Tomato plants growing with each of the *Trichoderma* strains. **(E)** Tomato plants infected with *R. solani* AG2. **(F–H)** Tripartite interaction between tomato plants, *R*. *solani* and each of the *Trichoderma* strains. Fresh weight **(I)**, primary root **(J)**, and stem **(K)** length of tomato plants after 8 weeks of interaction with the pathogen and the *Trichoderma* strains. Data from two independent experiments were analyzed by one-way ANOVA. Letters indicate statistically differences between treatments and the plants growing alone.

First, we analyzed the effect that the presence of the *T. atroviride* strains exerts on the tomato growth. We observed that, after 12 weeks, tomato plants that grew in the presence of the *Tal6-*OE1.1 strain had wider and longer root system and stem, compared to plants growing alone or in the presence of the wild type or *Tal6-*Δ4.2 mutant strains (Figures 8A–D,J,K). The same pattern was observed for fresh plant weight, plants that interacted with the OE strain had an increased weight (Figure 8I). This result suggests that the overexpression of *Tal6* has a beneficial effect over the tomato plant health.

The presence of *R. solani* AG2 has a negative effect over the plant health, evidenced by a loss of root and stem length, and a diminishing in fresh weight (Figures 8E,I–K). At the same time, we performed a tripartite interaction between tomato plants, each *Trichoderma* strain and *R. solani* AG2 to test if the overexpression of *Tal6* has also a beneficial effect over the plant when it is affected by a pathogen (Figures 8F–H).

Most of the plants interacting with either the wild type strain or the *Tal6-*OE1.1 strain survived the infection by *R. solani* AG2 (Table 1). In addition, in the surviving plants challenged with the pathogen and in interaction with the wild type or the *Tal6-*OE1.1 strain, we observed a significant increase in root and stem length and fresh weight when compared to the control condition, and to the plants interacting only with the pathogen (Figure 8J). We also observed a significant increase in fresh weight, stem and root length in the plants that were in interaction with both the pathogen and the *Trichoderma* strains, compared to the plants growing with the pathogen alone (Figures 8I–K).

**TABLE 1:** Plant survival percentage during the tripartite interaction between tomato, *R. solani* and each of the *T. atroviride* strains.

**Treatments/Number of plants**	**Percentage of survival (%)**
Ctrl. Tomato/16	93.75
*T. atroviride* WT-Tomato/16	100
*T. atroviride* OE1.1-Tomato/16	100
*T. atroviride* Δ4.2-Tomato/16	81.25
*R. solani* AG2-Tomato/16	25
*T. atroviride* WT-*R. solani* AG2-Tomato/24	45.83
*T. atroviride* OE1.1-*R. solani* AG2-Tomato/24	70.83
*T. atroviride* Δ4.2-*R. solani* AG2-Tomato/24	29.17

These results suggest that the protein Tal6 improves *Trichoderma* fitness to associate with the plants, exerting better overall health, and allowing *Trichoderma* to control more efficiently the pathogen when it infects the plants.

## Discussion

Plants can interact with the soil microbiota through the recognition of MAMPs, which activate a signaling cascade leading to physiological changes and the synthesis of compounds that participate in plant defense, such as phytoalexins, chitinases and other antimicrobial compounds. Some of those MAMPs are fragments of GlcNAc derived from the fungal cell wall (Stacey and Shibuya, 1997; Sánchez-Vallet et al., 2015). Several fungi have developed strategies that allow them to evade plant defense systems, making them successful pathogens (Toruño et al., 2016). This evasion of the defense involves the participation of effectors with LysM domains, which has been mainly characterized in pathogenic fungi with diverse lifestyles (De Jonge et al., 2010; Marshall et al., 2011; Mentlak et al., 2012; Takahara et al., 2016), including entomopathogens (Cen et al., 2017).

We confirmed the expression levels of four of the six genes with LysM domains from *T. atroviride* reported by Guzmán-Guzmán et al. (2017), in the presence of *A. thaliana*, observing an up-regulation only of *Tal2a* and *Tal6*. Genes from which, we did not detect any expression may need different conditions, for example, the presence of another plant species that could activate its transcription. We found that *Tal6*, besides being involved in the interaction with *Arabidopsis*, is also participating in the interaction with other fungi, as shown in our results of antagonism. Our results showed that *Tal6* expression is induced at times T2 and T3 (during and after contact, respectively) of interaction with *R. solani*. Gruber et al. (2011), reports that the genes *Tal1*, *Tal2*, *Tal4*, *Tal5*, and *Tal6* were expressed when *T. atroviride* was cultured in the presence of the phytopathogen *B. cinerea*. Guzmán-Guzmán et al. (2017) also reported that in confrontation with *R. solani* AG2 and AG5, *Tal2a* gene expression is induced. Therefore, it is possible that the *Tal1* and *Tal4* genes that we could not detect in the interaction with the plant could have a role in mycoparasitic interactions.

To better understand the function of the effector Tal6, we carried out several experiments to determine which of the proposed functions for LysM effectors could be more suitable to it (Seidl-Seiboth et al., 2013). Considering together the results of the experiments related to the susceptibility of the mycelium to chitinases, the antagonism and the *in vitro* activity of chitinases, we can propose that Tal6 is an effector that participates through two mechanisms, both related to the binding to chitin. On the one hand, binding to complex chitin in the hyphae, protecting them from the lytic attack and, on the other hand, hijacking GlcNac oligomers, which would function as MAMPs, attenuating the level of plant defense. Structural analysis of the LysM effector Ecp6 from *C. fulvum* revealed that the first and third LysM domains bind chitooligosaccharides with ultra-high affinity through the dimerization of intramolecular LysM, while the second domain shows low affinity to the fungal cell wall. However, both binding sites are capable of suppressing immune responses triggered by chitin (Sánchez-Vallet et al., 2013). It is unknown whether the effectors with only two LysM domains (Slp1, ChELP1, ChELP2, Vd2LysM), show a coordinated action regulating chitin binding. These data suggest that the number of LysM domains is not an important factor in determining the function of the protein. There are no reports about the specific role of *T. atroviride* cell wall chitin as a MAMP. Navazio et al. (2007), reported that a mixture of secreted metabolites of *T. atroviride* activated the defense responses in plant cells, this mixture includes proteins, peptides, antibiotics and oligosaccharides, among them, the chitin derivates could be present. Additionally, *T. atroviride* culture filtrates, which may contain remnants of fungal cell walls and other molecules, activated the expression of defense-related genes in cell cultures of *Vitis vinifera* (Mutawila et al., 2017). Moreover, in other symbiotic interactions, is known that arbuscular mycorrhiza secrete chitin-derived molecules, which stimulate the formation of mycorrhizal arbuscules in plants (Genre et al., 2013). These reports, as well as our results suggest that *T. atroviride* cell wall chitin could acts as MAMP as the same way that chitin in other pathogenic and non-pathogenic fungi, however, an experimental work aimed at elucidating this role should be done.

To support the fact that Tal6 could work through the two aforementioned mechanisms, a bioinformatic analysis was performed with other LysM domains from previously reported effectors, to elucidate whether the LysM domains in Tal6 are specific for a function. We found that domains M4, M5, M6, and M7 were grouped together and closely related to effector PeLysM2 from *P. expansum*, suggested to bind to cell wall chitin (Levin et al., 2017). Whereas motifs M1, M2, and M3 appeared separated from the other Tal6 motifs, and are related to the motifs M1 and M2 from effectors Blys2 and Blys5 from *B. bassiana*, respectively which bind cell wall chitin and sequester GlcNAc oligomers (Bolton et al., 2008; Cen et al., 2017). Seidl-Seiboth et al. (2013) proved the biological function of Tal6 domains M4-M7, in binding complex chitin, as we found in our biochemical experiments that the effector Tal6 in the *Tal6-*OE1.1 strain has affinity to chitin in the cell wall, to the chitotriose (Ch)_3_ present in the substrate 4-MU(Ch)_3_ and to GlcNAc monomer. Taken together, these results suggest that Tal6 domains M1-M3 may be responsible for the sequestering of GlcNAc monomers, meanwhile, domains M4-M7 could be responsible for protecting the hyphae by binding complex chitin in the cell wall.

Levin et al. (2017), generated mutant strains for *P. expansum* effectors (PeLysM1, PeLysM2, PeLysM3, and PeLysM4), finding that the mutation of PeLysM1, PeLysM2, and PeLysM4, did not affect growth and development, whereas the deletion of PeLysM3 did alter growth processes, like germination of spores. Seidl-Seiboth et al. (2013) also proposed that Tal6 could have a different function, being more related to fungal development, specially to the germination of conidia; since they observed inhibition of germination of conidia from *Trichoderma* spp. when adding the purified protein, and total inhibition at high protein concentrations of 1.8 μM, but no inhibition was observed in other fungi. We did not observe differences in the germination processes in any of the *Trichoderma* strains tested, it is possible that overexpressing strains do not produce enough protein to exert the negative effect on germination.

Plants can perceive chitin-derived MAMPs, activating a signaling cascade that leads to the production of defense molecules such as chitinases. Pathogen LysM effectors also hijack those GlcNAc units, avoiding detection by the plant receptors. Our results suggest that Tal6 may be interacting with GlcNAc oligomers released during the growth of *Trichoderma*, masking its presence before plant membrane receptors could perceive them. This was demonstrated by the *Arabidopsis* plants that were co-cultivated with the *Tal6-*OE1.1 strain, where the expression of two genes related to the production of chitinases was affected, showing that this non-pathogenic fungus could be using this effector as part of its evasion mechanisms for plant defense.

LysM effectors have been mainly studied in pathogenic systems and their function is related to virulence, preventing the triggering of the plant defense system. It is possible that Tal6 participates modulating the perception of the fungus by the plant, in such way that it can be established in the roots without being attacked or fully detected at the beginning of the interaction; in fact, the expression of *Tal6* is induced even before physical contact between the roots and the mycelium. Even though the additional amount of Tal6 in the overexpressing strain decrease the plant defense response, the beneficial *Trichoderma*-plant crosstalk is maintained, so the plants that were treated with the OE strain, showed better root and stem development, compared to the plants treated with the mutant and wild strain. These results suggest that, if Tal6 masks the fungus thus avoiding the initial perception by the plant, it is possible that *Trichoderma* could colonize better the plant roots, providing plant compounds that the fungus can also produce, such as phytohormones and others not yet identified (Guzmán-Guzmán et al., 2019) and that this is reflected in the increased growth of the plants treated with the *Tal6-*OE1.1 strain. This greater colonization is controlled by the plant, which could activate some other regulatory processes that do not depend on chitinases.

The confrontations between the strains of *Trichoderma* and several phytopathogens showed that the *Tal6-*OE1.1 strain has a greater antagonistic capacity. The results obtained from the tripartite interaction, suggest that the overexpression of Tal6 protects slightly more tomato plants, before the attack from *R. solani*, probably due to the combined action of positively stimulating the plant and limiting the growth of the pathogen. Resembling to this interpretation, Hermosa et al. (2013) integrated the participation of *Trichoderma* and proteins with LysM domains in two events related with their association with plants. On the one hand, they facilitate the colonization of the plant, by sequestering chitin, damping the plant defense response. On the other hand, as biocontrol agents, the micotrophic activity of *Trichoderma* chitinases release chito-oligosaccharides from the cell walls of phytopathogens, contributing to the induction of defense.

To our knowledge, this work is the first to functionally characterize a LysM effector, involved in a beneficial plant-fungus interaction, which suggests that the mechanisms used by fungi to remain inside the plant tissue, as endophytes are very similar to those of phytopathogens, with the difference being in the resulting effect of other compounds such as virulence factors. We found that the gene *Tal6* encodes an effector involved in mechanisms of colonization when *T. atroviride* interacts with plants, by sequestering chitin-derived compounds and by protecting the chitin present in the fungal cell wall, which may favor the establishment of *Trichoderma* in its hosts and conferring protection to the plant when encountered with phytopathogenic fungi.

## Data Availability Statement

The datasets generated for this study are available on request to the corresponding author.

## Author Contributions

YR-C and CR-V contributed equally to this work. YR-C, CR-V, PG-G, VO-M, and JV-C designed the experiments. YR-C, CR-V, and JM-S performed the experiments. YR-C, CR-V, and VO-M analyzed the data. PG-G, YR-C, and VO-M wrote the manuscript. All authors revised and approved the final version of the manuscript.

## Conflict of Interest

The authors declare that the research was conducted in the absence of any commercial or financial relationships that could be construed as a potential conflict of interest.

## Footnotes

1 https://weblogo.berkeley.edu/

2 https://idtdna.com

3 https://genome.jgi.doe.gov
