# Supplementary material for: Tal6 From Trichoderma atroviride Is a LysM Effector Involved in Mycoparasitism and Plant Association
Source: Front Microbiol. 2019 Sep 25;10:2231. doi: 10.3389/fmicb.2019.02231 (PMC6773873; doi:10.3389/fmicb.2019.02231)
Supplement: Supplementary file 1 [file Presentation_1.PPTX]

## Slide 1
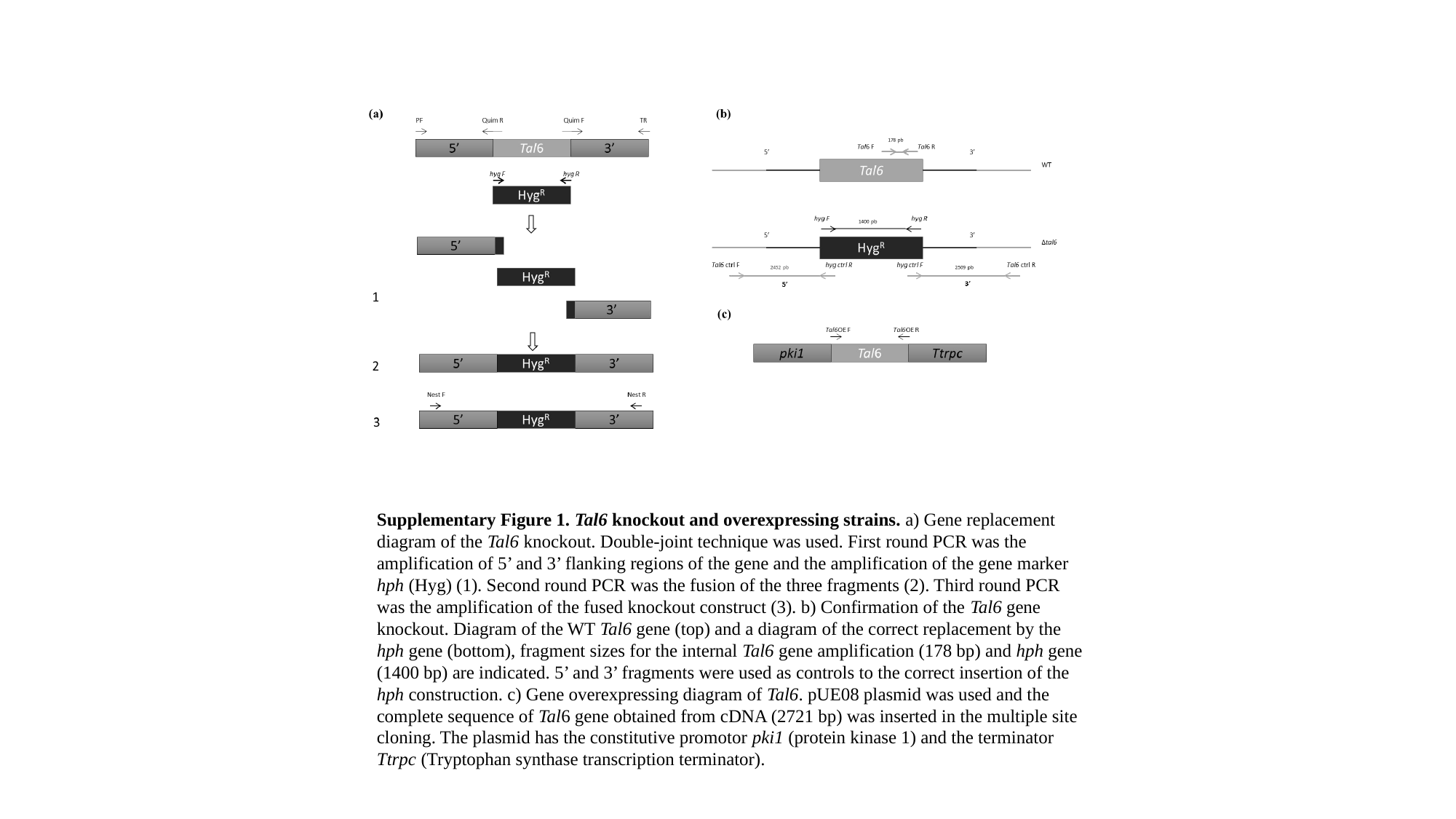

Supplementary Figure 1. Tal6 knockout and overexpressing strains. a) Gene replacement diagram of the Tal6 knockout. Double-joint technique was used. First round PCR was the amplification of 5’ and 3’ flanking regions of the gene and the amplification of the gene marker hph (Hyg) (1). Second round PCR was the fusion of the three fragments (2). Third round PCR was the amplification of the fused knockout construct (3). b) Confirmation of the Tal6 gene knockout. Diagram of the WT Tal6 gene (top) and a diagram of the correct replacement by the hph gene (bottom), fragment sizes for the internal Tal6 gene amplification (178 bp) and hph gene (1400 bp) are indicated. 5’ and 3’ fragments were used as controls to the correct insertion of the hph construction. c) Gene overexpressing diagram of Tal6. pUE08 plasmid was used and the complete sequence of Tal6 gene obtained from cDNA (2721 bp) was inserted in the multiple site cloning. The plasmid has the constitutive promotor pki1 (protein kinase 1) and the terminator Ttrpc (Tryptophan synthase transcription terminator).
