# Supplementary material for: Tal6 From Trichoderma atroviride Is a LysM Effector Involved in Mycoparasitism and Plant Association
Source: Front Microbiol. 2019 Sep 25;10:2231. doi: 10.3389/fmicb.2019.02231 (PMC6773873; doi:10.3389/fmicb.2019.02231)
Supplement: Supplementary file 2 [file Presentation_2.PPTX]

## Slide 1
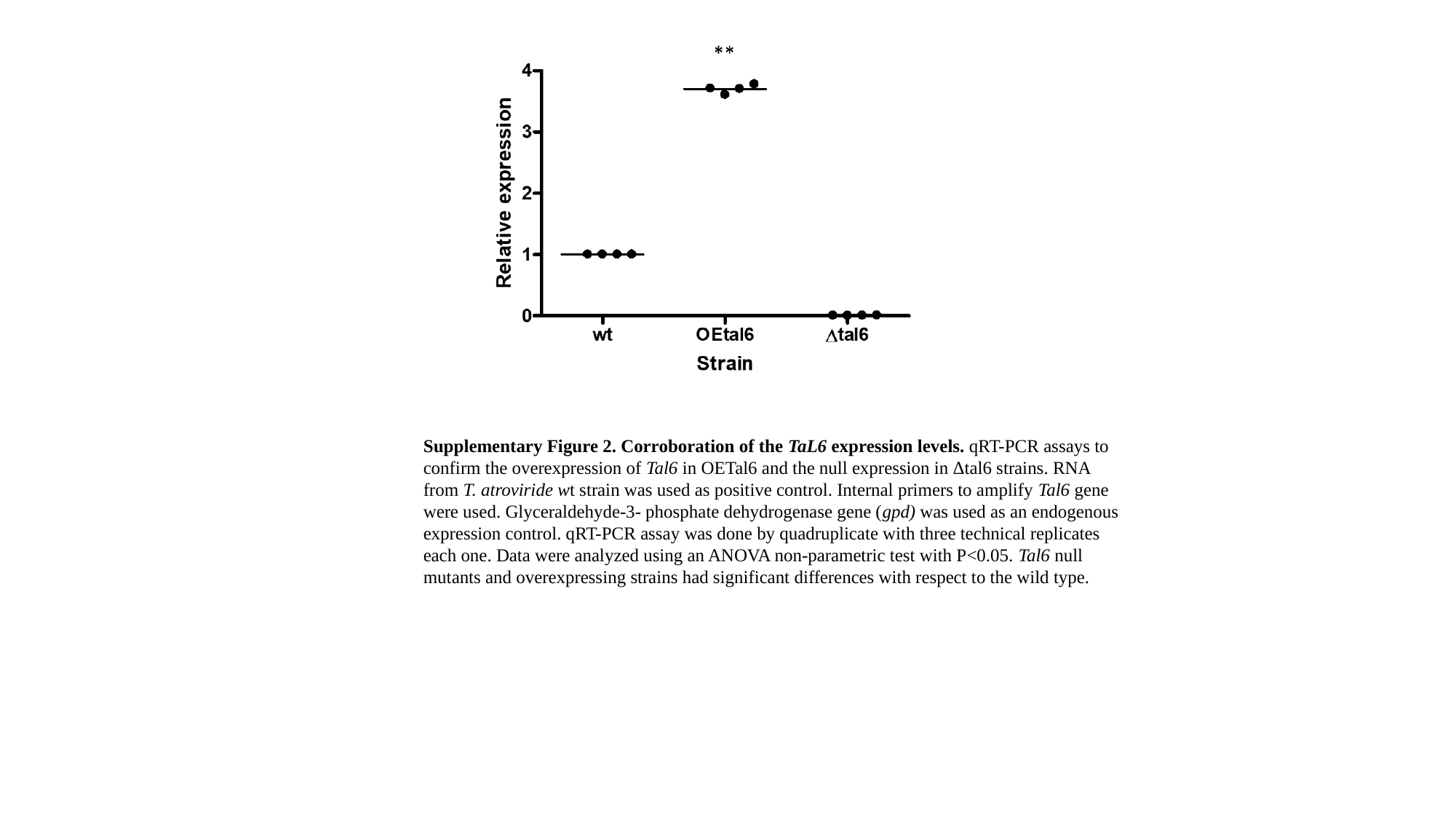

Supplementary Figure 2. Corroboration of the TaL6 expression levels. qRT-PCR assays to confirm the overexpression of Tal6 in OETal6 and the null expression in Δtal6 strains. RNA from T. atroviride wt strain was used as positive control. Internal primers to amplify Tal6 gene were used. Glyceraldehyde-3- phosphate dehydrogenase gene (gpd) was used as an endogenous expression control. qRT-PCR assay was done by quadruplicate with three technical replicates each one. Data were analyzed using an ANOVA non-parametric test with P<0.05. Tal6 null mutants and overexpressing strains had significant differences with respect to the wild type.
