# Supplementary material for: Tal6 From Trichoderma atroviride Is a LysM Effector Involved in Mycoparasitism and Plant Association
Source: Front Microbiol. 2019 Sep 25;10:2231. doi: 10.3389/fmicb.2019.02231 (PMC6773873; doi:10.3389/fmicb.2019.02231)
Supplement: Supplementary file 3 [file Presentation_3.PPTX]

## Slide 1
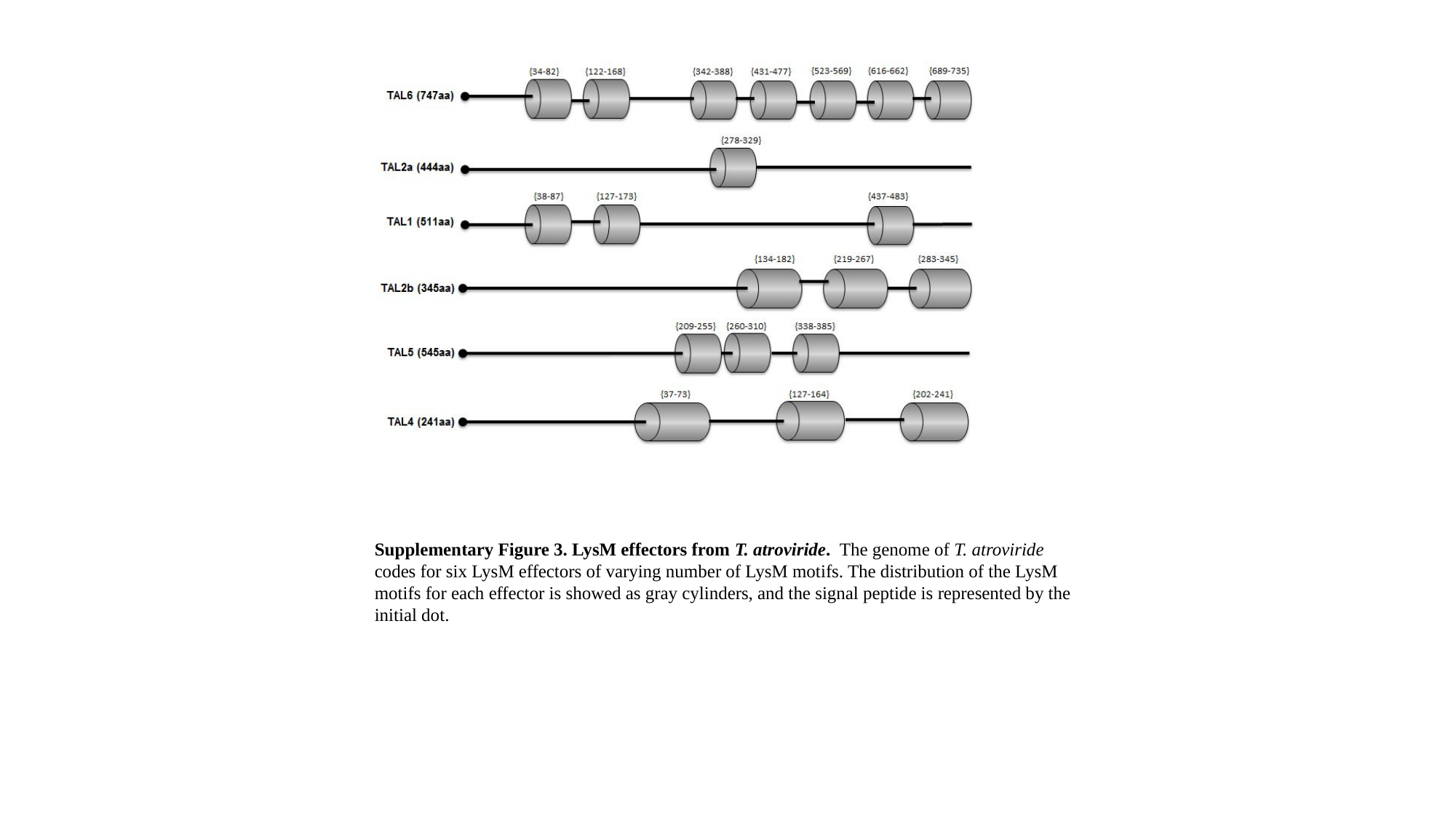

Supplementary Figure 3. LysM effectors from T. atroviride. The genome of T. atroviride codes for six LysM effectors of varying number of LysM motifs. The distribution of the LysM motifs for each effector is showed as gray cylinders, and the signal peptide is represented by the initial dot.
