# Supplementary material for: Tal6 From Trichoderma atroviride Is a LysM Effector Involved in Mycoparasitism and Plant Association
Source: Front Microbiol. 2019 Sep 25;10:2231. doi: 10.3389/fmicb.2019.02231 (PMC6773873; doi:10.3389/fmicb.2019.02231)
Supplement: Supplementary file 4 [file Presentation_4.PPTX]

## Slide 1
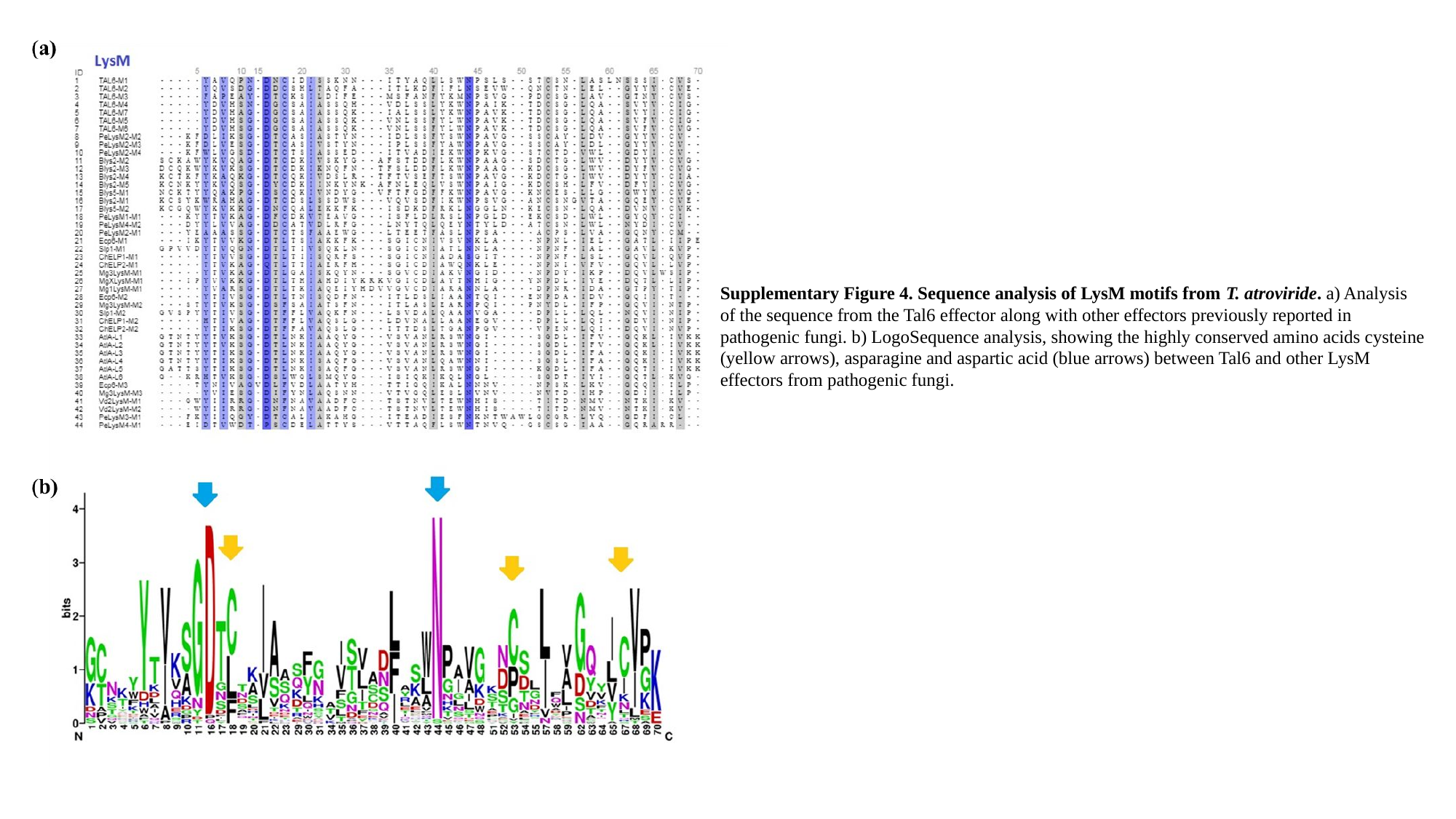

Supplementary Figure 4. Sequence analysis of LysM motifs from T. atroviride. a) Analysis of the sequence from the Tal6 effector along with other effectors previously reported in pathogenic fungi. b) LogoSequence analysis, showing the highly conserved amino acids cysteine (yellow arrows), asparagine and aspartic acid (blue arrows) between Tal6 and other LysM effectors from pathogenic fungi.
